# Improved in-vivo cardiac DTI using optimal b-values

**DOI:** 10.1186/1532-429X-16-S1-O27

**Published:** 2014-01-16

**Authors:** Andrew D Scott, Pedro Ferreira, Sonia Nielles-Vallespin, Laura-Ann McGill, Philip J Kilner, Dudley J Pennell, David Firmin

**Affiliations:** 1 Cardiovascular Biomedical Research Unit, The Royal Brompton Hospital, London, UK; 2 National Heart and Lung Institute, Imperial College, London, UK; 3 National Heart, Lung and Blood Institute, National Institute of Health, Bethesda, Maryland, USA

## Background

There has been much recent interest in the microstructural information available using cardiac diffusion tensor imaging (cDTI)[1-3]. cDTI measures signal loss between a reference (b0) and a diffusion weighted image. The signal loss is caused by both diffusion and other sources of intravoxel incoherent motion, such as microvascular perfusion. By applying diffusion weighting to the reference image (bref), the microvascular perfusion component, which has a high apparent diffusion coefficient could be eliminated allowing a measurement of diffusion alone[4]. However, in order to provide a sufficient difference in signal intensity, the amount of diffusion encoding (b-value) must be higher than in previous cDTI studies. We compare mean diffusivity (MD) and fractional anisotropy (FA) derived from cDTI acquired with b-values between b = 50 and b = 950 smm^-2 ^and separate diffusion from perfusion.

## Methods

cDTI was performed in 10 healthy subjects (7 male, age 23-57, Siemens Skyra) using the stimulated echo single shot EPI with monopolar diffusion encoding sequence, described previously[1]. A single short axis slice in the mid-ventricle was imaged at 2.8 × 2.8 × 8 mm^3 ^with 8 averages and 6 directions (+b0) at b = 50,150,350,550,750,950 smm^-2^. Pixel wise diffusion tensors were calculated using each b-value with b0 and also using all possible b-values as bref (e.g. b = 750 vs. bref = b0,50,150,350,550 smm^-2^). MD, FA and helical angle (HA) maps were derived in each case. For each subject the average diffusion weighted signal (averaged over all directions) at each b-value was calculated in the left ventricle.

## Results

**Figure 1:**
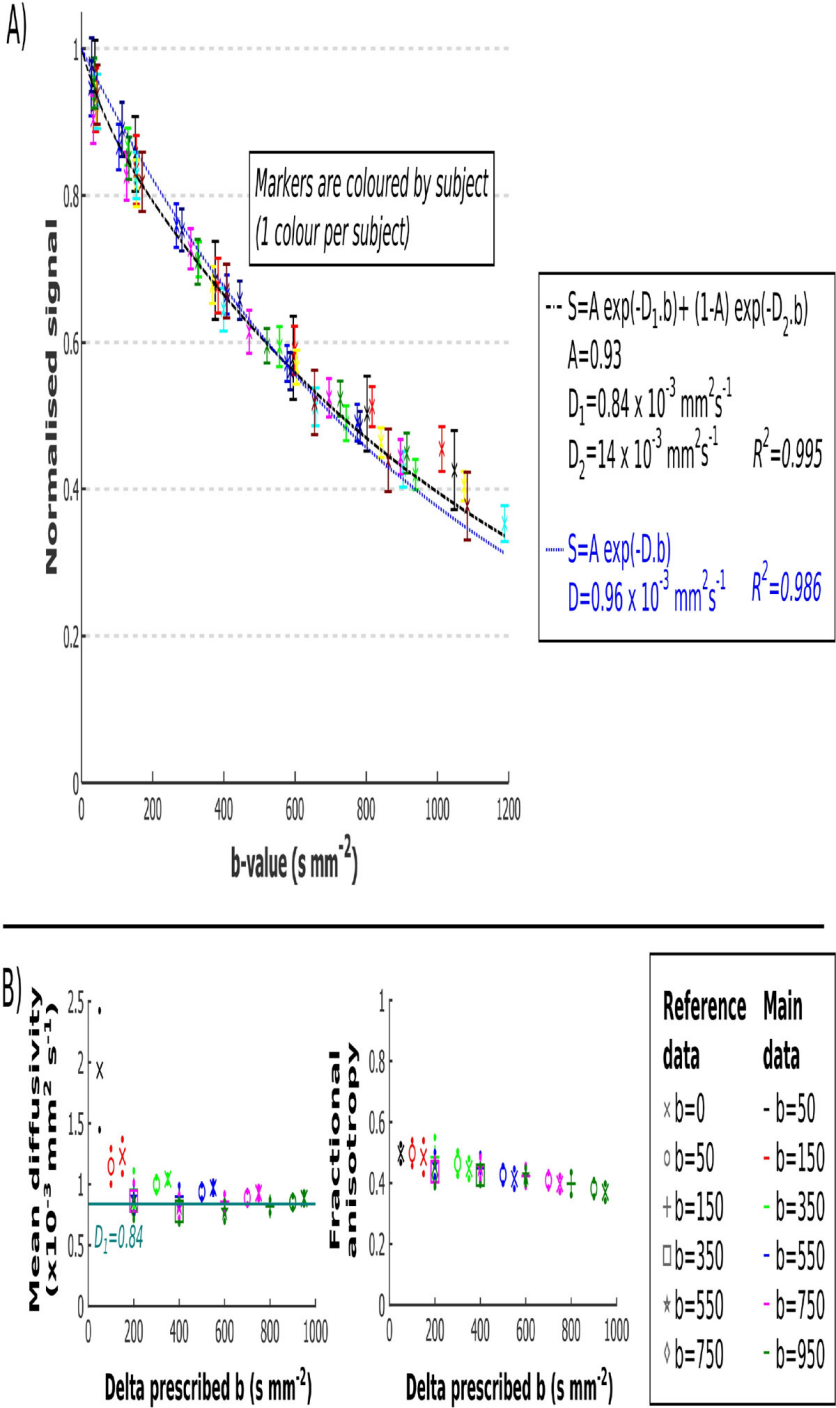
**Normalised signal intensity (A) and derived cDTI parameters (B) plotted with b-value**. Mono- and bi-exponential models are fitted to the normalised signal intensity vs. b-value corrected for heart rate (A). Mean (± SD as small dots) MD and FA are plotted for every possible combination of b-value (marker colour) and bref (marker type) (B).

**Figure 2:**
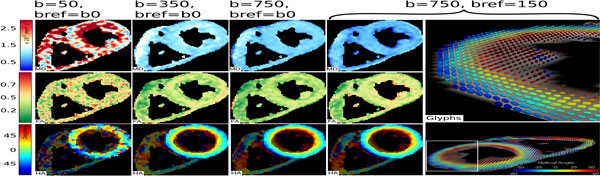
**Example cDTI parameter maps for 4 pairs of diffusion weightings**. For the b-value pairing selected as optimal (b = 750 vs. bref = 150 smm^-2^), superquadratic glyphs (right hand column) describing the full diffusion tensor in each pixel are also shown in whole short axis slice (lower) and in the anterolateral wall of the left ventricle (upper).

Figure 1A shows the signal loss in the left ventricle with b-value. A bi-exponential fit (fitted to b < 1000 smm^-2 ^to avoid the noise floor), with diffusion (D_1_) and microvascular perfusion (D_2_) components matches the data more closely than the standard mono-exponential model (R^2 ^= 0.995 vs. 0.986). By b = 150 smm^-2^, D_2 _contributes approximately 1% of the signal. Figure 1B gives mean MD and FA calculated using all pairs of b-values. With increasing bref the MD reduces and FA increases. MD is closest to D_1 _with b = 750 vs. bref = 150 smm^2^. Figure 2 shows example cDTI parameter maps calculated using 4 pairs of b-values.

## Conclusions

Increasing the b-value used in cDTI results in smoother MD and FA maps with less variation between subjects. Diffusion can be isolated from microvascular perfusion using a diffusion weighted reference images, which reduces the dependence of MD and FA on the b-value.

## Funding

This work was performed at the NIHR funded Cardiovascular Biomedical Research Unit at The Royal Brompton Hospital.
